# Thermosensitive Liposomes for Gemcitabine Delivery to Pancreatic Ductal Adenocarcinoma

**DOI:** 10.3390/cancers16173048

**Published:** 2024-09-01

**Authors:** Cesar B. Aparicio-Lopez, Sarah Timmerman, Gabriella Lorino, Tatiana Rogers, Marla Pyle, Tej B. Shrestha, Matthew T. Basel

**Affiliations:** 1 Department of Anatomy and Physiology, College of Veterinary Medicine, Kansas State University, Manhattan, KS 66506, USA; cesar03@vet.k-state.edu (C.B.A.-L.); sarahtimmerman@vet.k-state.edu (S.T.); glorino@vet.k-state.edu (G.L.); mpyle@vet.k-state.edu (M.P.); 2 Department of Electrical and Computer Engineering, Kansas State University, Manhattan, KS 66506, USA; tsrogers@ksu.edu; 3 Nanotechnology Innovation Center of Kansas State (NICKS), Kansas State University, Manhattan, KS 66506, USA; tbs3@vet.k-state.edu

**Keywords:** liposomes, thermosensitive, controlled release, gemcitabine, pancreatic cancer

## Abstract

**Simple Summary:**

Pancreatic cancer is one of the most deadly forms of cancer. Current treatment options often fail because too little of the chemotherapy gets into the cancer. Hyperthermia, or heat treatment, has shown some promise in treating pancreatic cancer and may make it more likely for the chemotherapy to enter into the cancer. This study aims to design liposomes that can increase the amount of chemotherapy reaching pancreatic cancer by targeting the liposomes with hyperthermia.

**Abstract:**

Treatment of pancreatic ductal adenocarcinoma with gemcitabine is limited by an increased desmoplasia, poor vascularization, and short plasma half-life. Heat-sensitive liposomes modified by polyethylene glycol (PEG; PEGylated liposomes) can increase plasma stability, reduce clearance, and decrease side effects. Nevertheless, translation of heat-sensitive liposomes to the clinic has been hindered by the low loading efficiency of gemcitabine and by the difficulty of inducing hyperthermia in vivo. This study was designed to investigate the effect of phospholipid content on the stability of liposomes at 37 °C and their release under hyperthermia conditions; this was accomplished by employing a two-stage heating approach. First the liposomes were heated at a fast rate, then they were transferred to a holding bath. Thermosensitive liposomes formulated with DPPC: DSPC: PEG2k (80:15:5, mole%) exhibited minimal release of carboxyfluorescein at 37 °C over 30 min, indicating stability under physiological conditions. However, upon exposure to hyperthermic conditions (43 °C and 45 °C), these liposomes demonstrated a rapid and significant release of their encapsulated content. The encapsulation efficiency for gemcitabine was calculated at 16.9%. Additionally, fluorescent analysis during the removal of unencapsulated gemcitabine revealed an increase in pH. In vitro tests with BxPC3 and KPC cell models showed that these thermosensitive liposomes induced a heat-dependent cytotoxic effect comparable to free gemcitabine at temperatures above 41 °C. This study highlights the effectiveness of the heating mechanism and cell models in understanding the current challenges in developing gemcitabine-loaded heat-sensitive liposomes.

## 1. Introduction

Pancreatic ductal adenocarcinoma (PDAC) is the most prevalent form of pancreatic cancer in the U.S. Its incidence continues to rise nationwide, accompanied by one of the lowest 5-year relative survival rates, hovering at approximately 10% [1,2,3,4]. According to the National Cancer Institute, pancreatic cancer in the U.S. constitutes roughly 3% of all cancer cases and contributes about 7% of all cancer-related deaths, making it the 4th deadliest cancer. Due to shifting demographics of aging, diabetes, and obesity, the incidence of PDAC is projected to double over the next decade [1]. The problem is further compounded by the lack of early detection methods and effective treatments for PDAC. Thus, it is poised to become the second deadliest cancer in the future [1,5,6].

The efficacy and prognosis of PDAC treatment are largely dependent upon the disease’s stage at the time of diagnosis and location in the pancreas, with 65% being located in the head of the pancreas [5,7]. The only potentially curative therapy available is surgical resection, followed by adjuvant chemotherapy or radiotherapy [8]. However, only 20% of PDAC patients present with resectable tumors, whereas 80% exhibit locally advanced, non-resectable tumors or distant metastases [9]. In cases where resection is not feasible or borderline resectability presents, systemic chemotherapy emerges as the primary treatment approach. This includes the administration of gemcitabine and capecitabine (nucleoside analogs) or 5-fluorouracil (pyrimidine analog) [10,11].

Gemcitabine is a chemotherapy drug that acts as an antimetabolite. Free gemcitabine is a weak base (pKa: 3.6) also known as 2′,2′-difluorodeoxycytidine (dFdC) and serves as a potent and well-tolerated chemotherapeutic agent [12]. Its minimal systemic toxicity stems from its swift conversion into the less potent difluoro-uridine derivative (dFdU), which is rapidly eliminated through renal processes, giving gemcitabine a plasma half-life of approximately 15 min [13,14,15]. The antitumor mechanism of action relies on the arrest of the cell cycle at the S-phase by inhibiting ribonucleotide reductase and DNA synthesis through the di- and tri-phosphorylated metabolites (dFdCDP and dFdCTP), respectively [12]. Nevertheless, due to fast clearance and degradation, high and frequent doses are required to achieve therapeutic effects, leading to an increase in adverse side effects [16].

Liposomes are spherical nanoparticles mainly consisting of phospholipids, featuring an aqueous inner core and an outer bilayer membrane [17,18,19]. Their exceptional biocompatibility and versatility in fabrication render them highly effective delivery vehicles for drugs [20]. Gemcitabine loading depends on the acidity of the liposome inner core, which protonates and positively charges gemcitabine, preventing it from crossing out of the lipid bilayer [21]. With a pKa of 3.6, gemcitabine requires a significantly lower pH compared to other drugs to be efficiently retained within the liposomes under normal physiological conditions. Thus, the increase of intraliposomal pH during processing could result in loss of gemcitabine and poor encapsulation efficiency.

Coating liposomes with polyethylene glycol (PEG) prevents plasma clearance by the reticuloendothelial system [18,22,23]. PEGylated liposomes show an increase in passive targeting due to longer circulation times; liposomes can infiltrate the interstitial space around the tumor due to leaky blood vessels and faulty lymph drainage (enhanced permeation and retention effect) [24,25,26]. Nonetheless, in highly desmoplastic tumors such as those found in pancreatic cancer, this effect is diminished due to physical barriers, such as fibrosis and positive interstitial pressure, which limit the number of molecules that can reach the tumor cells [27,28]. Therefore, exogenous stimuli such as heat (hyperthermia) have been used to increase permeability of the tumor fibrosis and induce angiogenesis; these effects favor the infiltration of particles and molecules to the tumor cells [29,30,31].

Mild hyperthermia (defined as tissue temperatures of 41–44 °C) increases blood perfusion and tumor permeability. The increased permeability can lead to the extravasation and retention of liposomes within the tumor region [32]. Similarly, hyperthermia induces the production of heat shock proteins due to heat stress. These proteins activate antigen-presenting cells, initiating an immune response to the tumor [33,34,35]. Despite the improved accumulation of liposomes accomplished by hyperthermia, gemcitabine is not biologically available because its slow release from the carrier keeps the concentrations below effective dosage [36]. Thermosensitive liposomes (TSLs) possess the capability to release encapsulated drugs in response to increases in temperature; this release is made possible by the reversible thermotropic transition properties of phospholipids [37,38]. The increase in temperature induces transition of the phospholipid phase from an ordered state to a disordered state, allowing the release of the payload [38]. Furthermore, the use of phospholipids with different transition temperatures allows researchers to shift the release temperature of liposomes, depending on the fractional percentages and transition temperatures of the phospholipids used [39]. The trigger release of TSLs occurs exclusively in tumor blood vessels where heat is applied, with minimal drug uptake by the tumor at normal temperatures [40]. The combination of TSLs and hyperthermia offers control of gemcitabine’s spatiotemporal release such that higher drug concentrations are released from the TSLs in the hyperthermic region.

Here, we propose to enhance gemcitabine’s targeting capabilities by encapsulating it in thermosensitive liposomes for more focused spatial–temporal release. In this study, we developed and characterized gemcitabine-loaded TSLs. We explored their behavior in pancreatic cancer cell models, particularly assessing how their cytotoxic effects vary with changes in temperature. This research aims to give insight into the potential of these liposomes for targeted use in pancreatic cancer treatment.

## 2. Materials and Methods

### 2.1. Materials

1,2-dipalmitoyl-sn-glycero-3-phosphocholine (DPPC; 850355); 1,2-distearoyl-sn-glycero-3-phosphocholine (DSPC; 850365); and 1,2-dipalmitoyl-sn-glycero-3-phosphoethanolamine-N-[methoxy(polyethylene glycol)-2000] (ammonium salt; PEG2k; 880160) were purchased from Avanti Polar Lipids, Alabaster, AL, USA. Oregon Green™, 514 Carboxylic Acid, Succinimidyl Ester (O6139), and 5(6)-carboxyfluorescein (404105000) were purchased from Thermo Fisher Scientific, Waltham, MA, USA. Gemcitabine hydrochloride (G0367) was purchased from TCI America, Portland, OR, USA. Dulbecco′s Phosphate Buffered Saline (PBS) (D8537) and RPMI-1640 medium (R8758) were purchased from Sigma Aldrich, St. Louis, MO, USA. Dulbecco′s Modified Eagle′s Medium–high glucose (D6429) and dimethyl sulfoxide (D128) were purchased from Fisher Scientific, Hampton, NH, USA. T-thermocouple (30AC8642) was purchased from Omega Engineering, Norwalk, CT, USA. MTT reagent (M922050) was purchased from RPI Corp., Mount Prospect, IL, USA.

### 2.2. Carboxyfluorescein Thermosensitive Liposome Manufacturing and Release

Temperature-sensitive liposomes (TSLs) were prepared by thin-film hydration followed by freeze–thaw cycling and heat extrusion at 50 °C. Phosphatidyl liposomes were composed of DPPC: DSPC: PEG2k with DSPC-PEG set at 5 mol% and DPPC = 100 − X-5 (PEG), DSPC = X; X: 30, 25, 20, 15, 10, or 5 mol%. Formulations are referred to by the percentage of DPPC in the formulation.

A hydrating solution of 50 mM carboxyfluorescein (CF) was prepared using a 250 mM sodium chloride base solution, followed with pH adjustment to physiological pH (7.4) using 0.1 M NaOH. Lipids at the selected ratios were mixed in chloroform to create a homogenous mixture and chloroform was slowly evaporated by air flush and heat cycles to create a thin lipid film in a 20 mL crystal vial. Next, the hydrating solution and the lipid film were heated up to 45 °C and the mix was vortexed to induce lipid film disruption and liposomal formation until no lipid residuals were seen on the vial. Subsequently, the lipid mix was subjected to 10 freeze–thaw cycles, using liquid nitrogen and a 45 °C hot water bath; this was followed by 10 cycles of heat extrusion at 50 °C through a polycarbonate membrane with a 100 nm pore diameter. Unencapsulated CF was removed by using a 20 cm × 1 cm chromatography column filled with Sephadex G-50 resin equilibrated with 1× PBS. Fractions containing CF-loaded liposomes were collected for later analysis.

CF-loaded liposomes were prepared as described above and each of the solutions was standardized to set the fluorescence within the linear range (5–12 mM CF) by diluting with PBS. A total of 100 µL of the liposome solution was then added to a well in a 96-well plate. To measure the temperature in real time, a 96-well plate was equipped with four T-type thermocouples and attached to a TC08 data logger. Two hot water baths were set up, one at 55 °C for temperature ramp-up and another for temperature holding (37, 42, and 45 °C). The liposomes were placed in the ramp-up bath until the desired temperature was reached, as measured by the thermocouple plate, and then transferred to the holding water bath. The plates were kept in the holding water bath for a set amount of time and then transferred to an ice-cold bath to prevent further CF release.

Plates were exposed to the holding temperature for 2, 5, 10, 15, 20, 25, or 30 min; the baseline is the fluorescence of the liposomes at room temperature. Fluorescence was read using Spectramax i3x.

### 2.3. Gemcitabine-Loaded Thermosensitive Liposome Manufacturing and Release

The liposome DPPC fraction that showed no significant release for 30 min at 37 °C was used for succeeding studies. A 2.5 mg/mL gemcitabine hydrochloride solution was prepared with 250 mM sodium chloride solution, and pH was adjusted to 2.8 with 0.1 M NaOH. The lipid film formation was created as described above. Lipid films were hydrated with the gemcitabine solution at 45 °C with vigorous mixing for 5 min to form gemcitabine liposomes. Freeze–thaw and heat extrusion cycles were performed as described above.

To evaluate gemcitabine loading in liposomes and the efficiency of drug release at various times and temperatures, several 100 μL aliquots were taken and diluted 10 times in 1X PBS: (I) a sample of liposomes after the freeze–thaw and heat extrusion cycles was kept to determine encapsulation efficiency; (II) a sample of Gemcitabine-loaded liposomes was kept at 37 °C for 30 min to measure base release of gemcitabine; (III) 0.1% Triton™ X-100 was added to liposomes at 60 °C for 30 min to quantify the total amount of gemcitabine in the final solution (TX-100); and (IV) samples heated at various temps with gentle mixing for 30 min to assess the release of gemcitabine from liposomes. A sample of liposomes was also added to 10% FBS/DMEM media to assess its stability in serum. Treated solutions were passed through Amicon^®^ Ultra spin filters (7kD MWCO) at 10,000 rpm at 4 °C for 15 min to extract free gemcitabine. A standard curve was created by dissolving gemcitabine in PBS. The absorbance at 270 nm of the resulting filtrate was measured in triplicate using a NanoDrop Spectrophotometer, and gemcitabine concentration in each liposome formulation was determined by interpolation to standard curves created for gemcitabine. Sample III was used to adjust the concentration of encapsulated gemcitabine to 250 μM, which was used for cell treatments.

### 2.4. Intraliposomal pH Measurements

The changes in intraliposomal pH are hypothesized to directly affect the gemcitabine retention. Oregon Green 514 was used to measure pH changes in the liposomes during preparation. This experiment was adapted from the work of DiCiccio [41]. A calibration curve was constructed by creating Oregon Green in DI water solutions at various pHs with a concentration of 34 µM Oregon Green; 150 µL of the Oregon Green solutions was added to a lipid film. The fluorescence of the solutions was measured with a SpectraMax i3X with an excitation wavelength of 488 nm; emission spectra were recorded from 513 nm to 700 nm. Spectra were normalized by dividing the respective wavelength fluorescence values by the fluorescence at peak emission (523 nm) to determine a pH-dependent emission peak. The emission intensity ratio of 523 nm/558 nm was then plotted against pH to produce a standard curve.

To measure pH changes in liposomes during processing, liposomes were prepared as described above using a hydration solution of 2.5 mg/mL gemcitabine (pH 3.2) with 34 µM Oregon Green 514. Free gemcitabine/Oregon Green was removed using the Sephadex G-50 resin column chromatography method described above. The liposomal mixture emission spectrum was read before and after column chromatography purification. Then, the results were compared to the calibration curve described above to determine the pH inside the liposomes.

### 2.5. Cell Culture

KPC cells were cultured in T25 flasks in Dulbecco’s Modified Eagle medium (DMEM) supplemented with 10% fetal bovine serum (FBS) and 1% penicillin–streptomycin. BXPC3 cells were cultured in T75 flasks in RPMI-1640 supplemented with 10% FBS and 1% penicillin–streptomycin. All cells were kept at 37 °C in a humidified 5% ${CO}_{2}$ incubator. Both cell lines were allowed to reach 90% confluency before being seeded into 96-well plates for experimental procedures. KPC cells were seeded at a density of 25,000 cells/cm^2^ and BXPC3 cells were seeded at 50,000 cells/cm^2^.

### 2.6. Cell Hyperthermia Treatment

**Figure 1:**
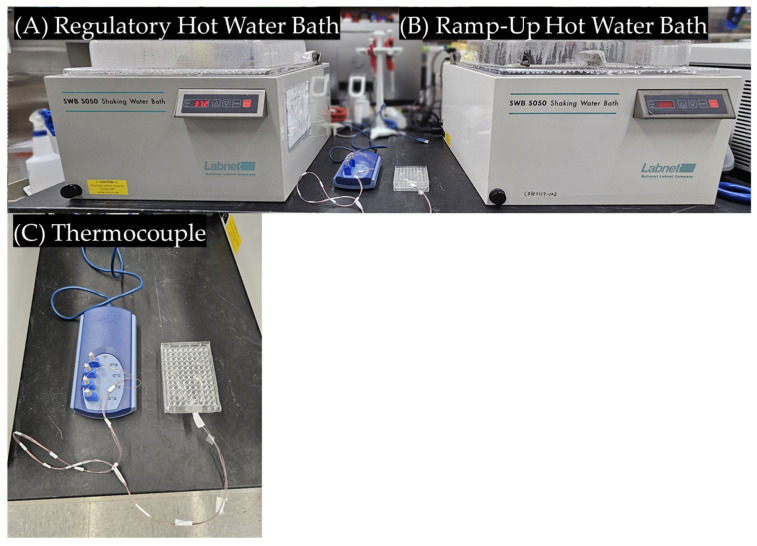
To improve the heating protocol and reduce the undesired release, a ramp-up water bath is used to increase the temperature at a rapid rate (**B**). The temperature of the cell is correlated to a homologous plate equipped with four thermocouples (**C**). Once the desired temperature is reached, the plates are transferred to the regulatory bath (**A**).

The 96-well plates seeded with KPC or BxPC3 cells were prepared as described above. A two water bath setup was used; the temperature changes were measured with a 96-well plate equipped with several T-thermocouples (Figure 1) [42]. First, a 55 °C water bath was used to rapidly increase the temperature to the desired set point. Once the desired temperature was reached, the 96-well plates were transferred to a second water bath set to the temperature desired for holding (37, 39, 41, 43, or 45 °C. The plate was divided into negative control (no treatment), positive control (unencapsulated gemcitabine), and experimental (encapsulated gemcitabine) groups (9 wells per group). Free gemcitabine or gemcitabine-TSLs (250 µM of gemcitabine) were added to DMEM or RPMI with no FBS or antibiotics for cell treatment. The probe plate and the cell-containing plate were placed in the 55 °C bath until the target temperature was reached, and then both plates were transferred to the temperature-holding bath. Plates were held in the holding bath for 20 min and then were removed from the water baths. Immediately, the media on the plates was removed and replaced with fresh 10% FBS-containing media. The plates were then returned to the incubator at standard environments (37 °C, 5% CO_2_, 95% humidity). After 24 h, the plates were treated with 100 μL of 0.5 mg/mL MTT reagent for 3 h. Then, 125 μL of DMSO was added to dissolve the MTT crystals formed, and the plates were incubated for another 2 h. The absorbance of the wells was then read at 570 nm with a reference at 650 nm using a BioTek Synergy™ H1 hybrid multi-mode microplate reader.

### 2.7. Statistical Analysis

Statistical analysis was conducted in GraphPad Prism 10. Two-way ANOVA with a Tukey’s multiple comparison test was used with α < 0.05. The Tukey *p* values are adjusted based on the number of replicates (see each analysis).

## 3. Results

### 3.1. Liposome Characterization

**Figure 2:**
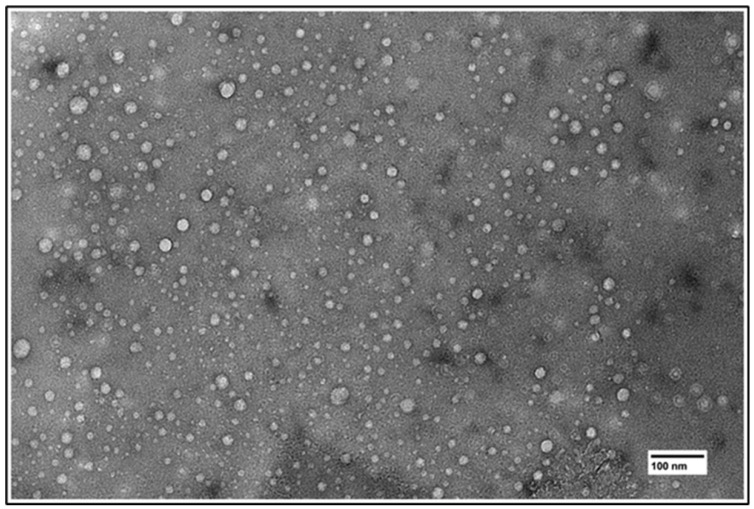
TEM image of synthesized liposomes.

Particle size and zeta potential in CF-loaded liposomes showed no clear differences in hydrodynamic size (HS), ranging from 114 nm to 126 nm (Table 1). The TEM images show a narrow size distribution (Figure 2). Likewise, gemcitabine-loaded thermosensitive liposomes had a HS of 120 ± 8 nm. Zeta potentials for CF-loaded and gemcitabine-loaded liposomes were approximately −2.1 ± 0.21 mV (Table 1).

### 3.2. Liposome Stability

**Figure 3:**
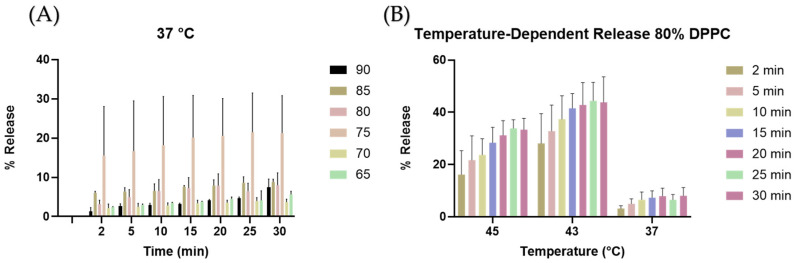
Release of CF as a function of time and temperature. (**A**) Release of CF at 37 °C versus time. (**B**) Temperature-dependent CF release from 80% DPPC fraction liposomes.

The stability of the liposomes was measured by finding the rate of release of CF from liposomes with different DPPC ratios over time; the fluorescence reading of each TSL prep at room temperature was used as the baseline fluorescence. The maximum release was determined by lysing the TSLs with 10% Triton-X. The baseline was subtracted from all fluorescence measurements. Then, the percentage release was calculated as the ratio of Triton-X to each temperature-dependent fluorescence measurement. Analysis showed the 80% DPPC liposomes to be the most stable with non-significant change in release percentage during 30 min at 37 °C (Figure 3A). The 80% DPPC liposomes were selected for later experiments. To determine whether the liposomes showed heat-dependent release, liposomes were exposed to constant-temperature baths and CF release was measured. The 80% DPPC liposomes loaded with CF demonstrated a trend toward maximal release at 43 °C with a maximum percent release of 53% (Figure 3B). There is a decrease in fluorescence at 45 °C; this change can be attributed to evaporation from the well and condensation into the plate lid; this causes the concentration of CF to increase due to the reduction of volume. At higher concentrations, CF self-quenches, effectively reducing fluorescence.

### 3.3. Encapsulation Efficiency

**Figure 4:**
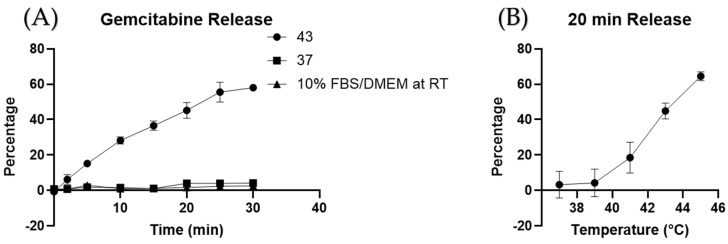
Gemcitabine release. Gemcitabine release is temperature-dependent (**A**). Gemcitabine-loaded TSLs under normal vs. hyperthermia conditions for 20 min (**B**).

Gemcitabine encapsulation efficiency for gemcitabine-loaded liposomes was determined. The Equation (1) for calculating encapsulation efficiency, EE, is:

(1) $$EE=\frac{S-F}{S}\times 100$$

where S is the initial gemcitabine concentration in the loading buffer and F is the final concentration in the loading buffer after loading. The loading efficiency measured for the gemcitabine-loaded liposomes was 16.9% ± 2%. Gemcitabine-loaded liposomes were also stable at 37 °C for 30 min and in 10% FBS/DMEM media, as determined by percent release versus time (Figure 4A). The maximum release temperature was determined to be between 41 °C and 42 °C (Figure 4B).

**Figure 5:**
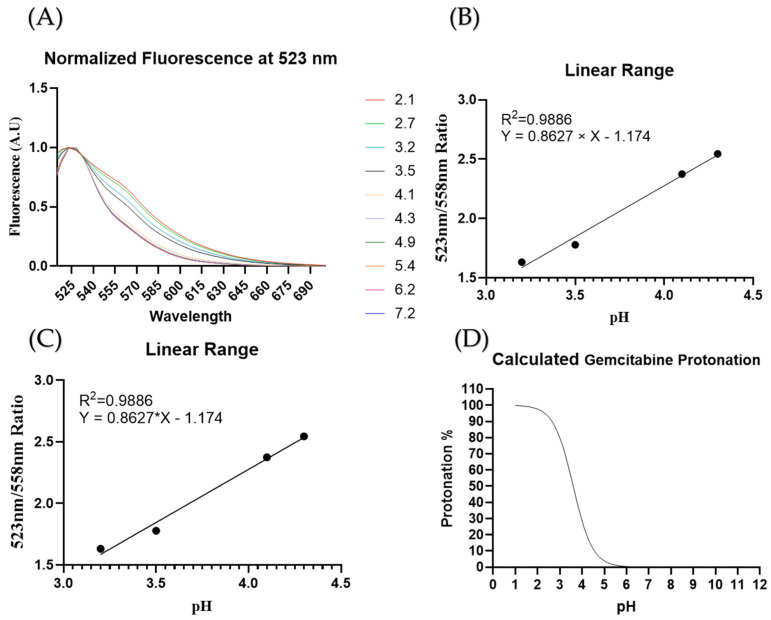
Oregon Green pH determination. (**A**) Normalization of signal to remove concentration variation. (**B**) pH vs. ratio plot. (**C**) pH–fluorescence linear range. (**D**) Calculated protonation percentages of gem.

To assess the encapsulation efficiency of gemcitabine relative to pH, changes in intraliposomal pH were determined by measuring the fluorescence shifts of Oregon Green. First, normalization against fluorescence at 523 nm of pH-dependent fluorescence showed a pH-dependent peak at 558 nm (green arrow, Figure 5A). A ratio between the pH-independent peak (red arrow) and the pH-dependent peak (green arrow) is plotted against pH, yielding a sigmoidal curve (Figure 5B). This yielded a fluorescence linear range between a pH of 3.2 and 4.3 (Figure 5C). Finally, the expected protonation percentage was calculated and plotted against pH values (Figure 5D). Using the fluorometric ratio calculation previously set, intraliposomal pH was calculated (Table 2). The 80% DPPC liposomal formulation shows a change of pH values after liposomes were processed with the Sephadex 50G resin chromatography column.

### 3.4. In Vitro Viability Studies

**Figure 6:**
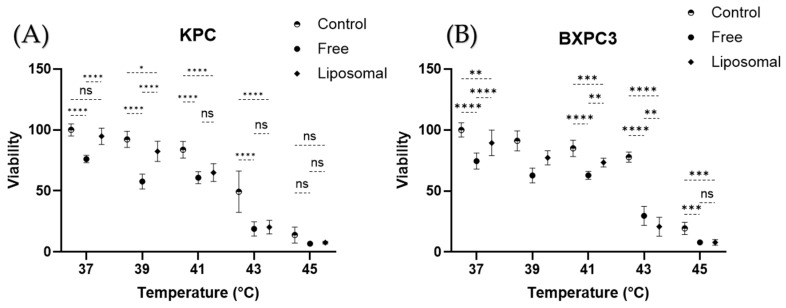
MTT cytotoxicity studies. (**A**) KPC cell line. (**B**) BXPC3 cell line. ns: *p* > 0.05; *: *p* ≤ 0.05; **: *p* ≤ 0.01; ***: *p* ≤ 0.001; ****: *p* ≤ 0.0001.

In vitro cytotoxicity assays were conducted with two cell line models (BxPC3 and KPC) using liposomal gemcitabine or a free gemcitabine concentration of 250 µM, simultaneous hyperthermia treatment (39, 41, 43, and 45 °C), and a treatment time of 20 min. In the KPC cell model (Figure 6A), a 37 °C control shows no significant difference when compared to the liposomal gemcitabine (*p* > 0.05), while there is a decline in cell viability of free gemcitabine against the untreated control (*p* < 0.05). When the temperature is increased to 41 °C, liposomal gemcitabine and free gemcitabine present no significant difference (*p* > 0.05), while the control group differs from the liposomal gemcitabine group (*p* > 0.05). On the other hand, the BxPC3 model (Figure 6B) shows significant differences amongst the three groups at any temperature; nevertheless, a similar temperature-dependent trend can be observed (Figure 6B).

## 4. Discussion

This study shows that gemcitabine-loaded thermosensitive liposomes that have transition temperatures in the mild hyperthermia range can be synthesized; this can be achieved by varying the fraction of phospholipids with different transition temperatures. The liposomes can be used to reduce off-target side effects and to narrow the treatment area to any hyperthermic region. These two characteristics allow the treatment of pancreatic cancer while sparing healthy tissue.

The lipid fractions used in this study yield liposomes of similar sizes and dispersity indexes (Figure 2 and Table 1). Thus, size was not a factor when choosing the DPPC fraction to be used in later studies. Similarly, the Z-potential of the liposomes was similar due to the same proportion of PEG (5% mol) being used in all formulations (Table 1). It is widely known that temperature-dependent release of drugs from thermosensitive liposomes depends on the transition temperature of the lipids being used. The transition temperature of DPPC is 41 °C, while that of DSPC is 55 °C [39]. Nevertheless, due to the geometrical interactions of the phospholipids, liposomes can burst release at temperatures below the lowest transition temperature. This is demonstrated with the 75% DPPC fraction in Figure 3A, which shows a large release variability at 37 °C. In addition, since the term “transition temperature” refers to the temperature where the membrane is most permeable [43], some release can be expected from all formulations at temperatures below the membrane transition temperature. Thus, despite most of the DPPC fractions demonstrating some release at 37 °C, the 80% fraction was chosen after statistical analysis showed no significant release changes over time (SI2).

Typically, liposomal cargo loading is done using hydrating solutions at physiological pH because the molecules of interest have a pKa larger than 7.4. However, because gemcitabine must be charged to be entrapped within the liposomes, the pH needs to be lowered to increase the protonation of gemcitabine and the resultant percentage of molecules entrapped. First, we added the gemcitabine to the hydrating solution while keeping the pH low in order to trap the gemcitabine molecules after liposome formulation. Encapsulation efficiency was used to assess the amount of gemcitabine entrapped by measuring the starting concentration of gemcitabine and the concentration after heat extrusion (Equation (1)). Usually, the release profile graph of a TSL shows a decrease in release after the transition temperature is reached [39]. However, due to the low concentration of gemcitabine used in our experiments, the low gemcitabine gradient across the liposomal gradient prevents the burst release of gemcitabine, causing a continued increase in release, as seen in Figure 4A,B. As mentioned, poor loading efficiency has characterized liposomal formulations of gemcitabine. Though the loading methods can improve loading efficiency, we hypothesize that the proton concentration is high at the hydration step and later increases during chromatography (Table 2). The reduction in concentration of both protons and payload (gemcitabine/Oregon Green) during the chromatography step can cause changes in absorbance and fluorescence. The use of a ratiometric graph allows us to overcome the loss of Oregon Green molecules (Figure 5A). Then, by using the lowest pH value within the linear range of a pH against fluorescence graph, 3.2 (Figure 5B,C), we assess the pH after liposomal purification (Table 2). Figure 5D predicts the protonation percentage of gemcitabine at a given pH. The protonation percentage can then be inferred to be the maximal amount of gemcitabine that can be kept inside a liposome. When comparing the protonation percentage at the calculated pH from Table 2, the expected protonation and the loading efficiency are close values. In short, we learned that there is a difference between pH pre- and post- column chromatography for unencapsulated gemcitabine.

Two cell lines, BXPC3 and KPC, were used to evaluate the killing capacities of gemcitabine in a temperature-dependent manner. An ideal gemcitabine-loaded TSL would have no effect on cell viability at temperatures below hyperthermia regardless of exposure time, thus maintaining viability like the negative control group. However, upon heat stimulation, the release of gemcitabine from liposomes would be expected to have effects like those of the free gemcitabine treatment. We compared the cytotoxic effect of gemcitabine-loaded TSLs against that of free gemcitabine. Hence, we limited the exposure of the cells to gem formulations to 20 min. Several studies use exposures of over 12 h, which does not emulate the conditions of the drug in the body, since gemcitabine has such a short plasma half-life [10,12]. Only the KPC cell line demonstrated a response to liposomal treatment similar to that expected of an ideal TSL. In addition, there are marked growth differences between the two cell lines. KPC cells are characterized by their high growth rate, while BXPC3 cells tend to reach confluency at a slower rate [44,45]. These metabolic states could strongly affect the response to chemotherapeutic agents, precluding correct release–cytotoxicity correlation comparisons. It is worth noting that pancreatic cancer is protected by physical barriers (fibrosis and hypovasculature), so that drug delivery depends mostly on the peripheral circulation of the tumor [28,46].

## 5. Conclusions

Gemcitabine-containing liposomes were successfully synthesized and were stable at temperatures below 41 °C but quickly released their contents above 43 °C. These liposomes may be able to be used in combination hyperthermia–gemcitabine treatments to increase delivery to the tumor while reducing systemic exposure to gemcitabine.

## Figures and Tables

**Table 1:** Size distribution and zeta potential for CF-loaded liposomes.

DPPC Content	Size (nm)	PDI	Z-Potential (mV)
65%	125.7 ± 2.5	0.058 ± 0.010	−1.89
70%	123.3 ± 3.1	0.092 ± 0.022	−2.31
75%	119.1 ± 3.6	0.040 ± 0.009	−2.17
80%	114.7 ± 3.5	0.083 ± 0.013	−1.82
85%	124.8 ± 3.4	0.049 ± 0.027	−2.24
90%	124.8 ± 3.2	0.052 ± 0.024	−1.97

**Table 2:** Liposome pH.

	Pre-Column	Post-Column
523/558	1.5	2.3
Calculated pH	3.2	4.2

## Data Availability

Data are contained within the article.
